# Supplementary material for: Integrated miRNA Changes in Canine Testis and Epididymis According to Age and Presence of Cryptorchidism
Source: Animals (Basel). 2023 Apr 18;13(8):1390. doi: 10.3390/ani13081390 (PMC10135127; doi:10.3390/ani13081390)
Supplement: Supplementary file 1 [file animals-13-01390-s001.zip › animals-2210909-supplementary.pdf]

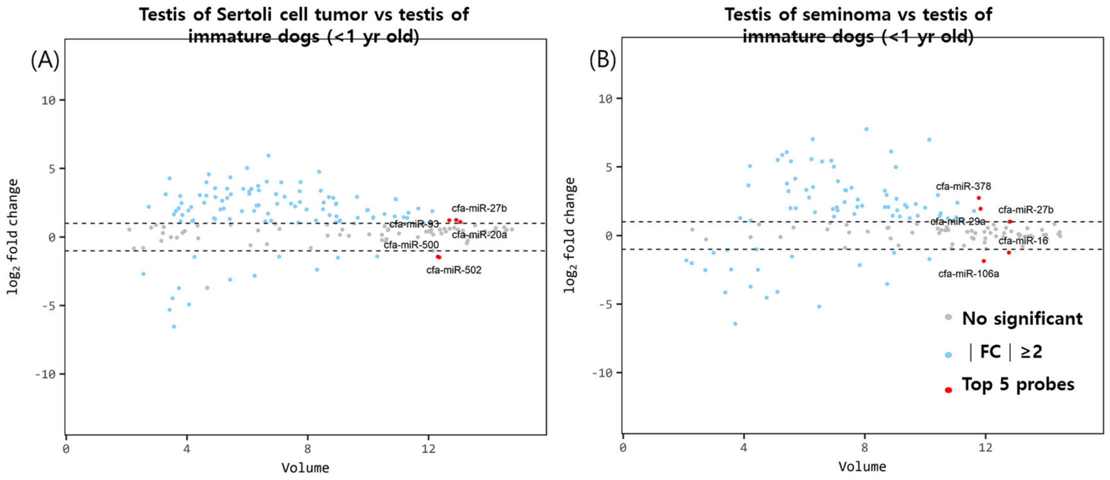

Figure S1. Volume plots of miRNAs in (**A**) Sertoli cell tumor and (**B**) seminoma compared with testis of age-matched dogs. The volcano plot illustrates the fold change and volume between healthy immature and testicular tumor dogs. The horizontal line indicates a 2-fold change difference. The red dots indicatethe top five significant miRNAs for comparisons. Sky color dots indicate significance above a 2-fold change difference without a p-value of less than 0.05. Cfa-miR = *Canis lupus familaris* microRNA. Gray dots signify no meaningful correlation between volume and fold change.

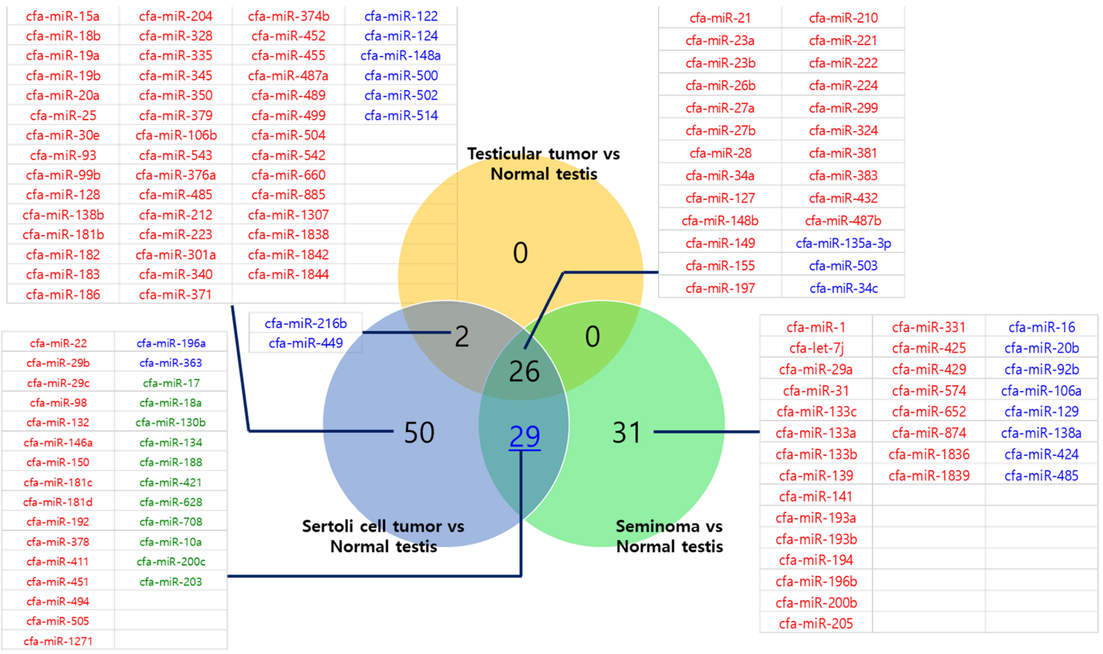

Figure S2. Venn diagram illustrating miRNA expression patterns in the three comparisons of testicular tumor (Sertoli cell tumor, Seminoma) and normal testis. Venn diagram representing differentially expressed miRNA observed in the comparisons among the three classes (normal, Sertoli cell tumor, and Seminoma). The numbers indicate the miRNA counts in the indicated area. The tables show the miRNAs identified by each comparison. Red indicates differentially upregulated miRNAs and blue indicates downregulation. MiRNAs indicated in green were upregulated in one comparison, which was shown to have

contradictory results in each comparator group, but were down regulated in the other comparison.
